# Quantitative SSTR-PET/CT: a potential tool for predicting everolimus response in neuroendoctine tumour patients

**DOI:** 10.2478/raon-2024-0032

**Published:** 2024-06-12

**Authors:** Homeira Karim, Michael Winkelmann, Freba Grawe, Friederike Völter, Christoph Auernhammer, Johannes Rübenthaler, Jens Ricke, Maria Ingenerf, Christine Schmid-Tannwald

**Affiliations:** Department of Radiology, University Hospital, LMU Munich, Munich, Germany; Department of Nuclear Medicine, University Hospital, LMU Munich, Munich, Germany; ENETS Centre of Excellence, Interdisciplinary Center of Neuroendocrine Tumours of the GastroEnteroPancreatic System at the University Hospital of Munich (GEPNET-KUM), University Hospital of Munich, Munich, Germany; Department of Internal Medicine 4, University Hospital, LMU Munich, Munich, Germany

**Keywords:** neuroendocrine tumors, SSTR-PET/CT, everolimus, response

## Abstract

**Background:**

This study aimed to assess ^68^Ga-DOTA-TATE (-TOC) PET/CT quantitative parameters in monitoring and predicting everolimus response in neuroendocrine tumor (NET) patients with hepatic metastases (NELM).

**Patients and methods:**

This retrospective analysis included 29 patients with 62 target lesions undergoing everolimus treatment and pre-therapy, and follow-up ^68^Ga-DOTA-TATE (-TOC) PET/CT scans. Response evaluation utilized progression-free survival (PFS) categorized as responders (R; PFS > 6 months) and non-responders (NR; PFS ≤ 6 months). Lesion size and density, along with maximum and median standardize uptake value (SUV) in target lesions, liver, and spleen were assessed. Tumor-to-spleen (T/S) and tumor-to-liver (T/L) ratios were calculated, including the tumor-to-spleen (T/S) ratio and tumor-to-liver (T/L) ratio (using SUVmax/SUVmax, SUVmax/SUVmean, and SUVmean/SUVmean).

**Results:**

PET/CT scans were acquired 19 days (interquartile range [IQR] 69 days) pre-treatment and 127 days (IQR 74 days) post-starting everolimus. The overall median PFS was 264 days (95% CI: 134–394 days). R exhibited significant decreases in Tmax/Lmax and Tmean/Lmax ratios compared to NR (p = 0.01). In univariate Cox regression, Tmean/Lmax ratio was the sole prognostic parameter associated with PFS (HR 0.5, 95% CI 0.28–0.92, p = 0.03). Percentage changes in T/L and T/S ratios were significant predictors of PFS, with the highest area under curve (AUC) for the percentage change of Tmean/Lmax (AUC = 0.73). An optimal threshold of < 2.5% identified patients with longer PFS (p = 0.003). No other imaging or clinical parameters were predictive of PFS.

**Conclusions:**

This study highlights the potential of quantitative SSTR-PET/CT in predicting and monitoring everolimus response in NET patients. Liver metastasis-to-liver parenchyma ratios outperformed size-based criteria, and Tmean/Lmax ratio may serve as a prognostic marker for PFS, warranting larger cohort investigation.

## Introduction

Neuroendocrine tumors (NET) are rare, with an incidence of 0.48 per 100,000 population.^1,2,3^ These tumors are often diagnosed at an advanced stage, frequently with liver metastases, limiting curative surgical options and shifting the focus of treatment towards symptom control and reducing tumor spread. Targeting and inhibiting the mTOR protein, which plays a role in the tumorigenesis of NETs of different origins, has emerged as a promising therapeutic strategy for NETs.^1,4^

Everolimus, an mTOR inhibitor, has shown effectiveness as a second-line therapy in advanced pancreatic NETs, prolonging progression-free survival (PFS) and improving overall survival.^1,5,6,7^ However, objective tumor response rates to are generally low, indicating that tumor growth stabilization rather than shrinkage is the primary outcome.^5,8^ Conventional response criteria based on tumor size change appear to be suboptimal for evaluating treatment response to targeted anti-cancer drugs in slow-growing tumors like NETs.^8,9,10^ However, it would be crucial to distinguish responders from non-responders early on in clinical practice and enhance the design of oncological studies by establishing a more precise definition of progression-free survival (PFS). The Choi criteria, which incorporate changes in tumor density on CT in addition to size, have demonstrated a better correlation with overall survival: A study by Solis-Hernandez analysed 107 patients with neuroendocrine tumors treated with sunitinib: They found out that Median progression-free survival (PFS) by RECIST and Choi were 11.42 (95 % confidence interval [CI], 9.7–15.9) and 15.8 months (95% CI, 13.9–25.7). In addition, PFS by Choi exhibited greater correlation with overall survival (OS) than PFS by RECIST and RECIST incorrectly estimated prognosis in 49.6%.^11^

PET/CT with ^68^Ga-labeled somatostatin analogues (SSA) (^68^Ga-DOTA-TATE, -DOTA-NOC and -DOTA-TOC) has potential to provide new imaging biomarkers for NETs: The majority of well to moderately differentiated neuroendocrine tumors (NETs) (80–95 %) overexpresses somatostatin receptors (SSTRs) on cell surfaces. PET/CT with ^68^Galabeled somatostatin analogues (SSA) (^68^Ga-DOTA-TATE, -DOTA-NOC and -DOTA-TOC) enables visualization of SSTRs and correlates with the histopathological expression of SSTRs^12,13,14^ and provides a comprehensive assessment of NETs, including their location, size, and metabolic activity.^15^ It is recommended for initial staging and follow-up of gastroenteropancreatic neuroendocrine tumors (GEP-NET) by the European Society for Medical Oncology Guidelines Working Group.^16^

However, there is limited research on functional imaging response criteria in patients receiving everolimus. Therefore, this study aims to assess the diagnostic reliability of ^68-^Ga-DOTA-TATE PET/CT to monitor and predict therapy response to everolimus in NETs.

## Patients and methods

### Patients

This retrospective study included consecutive patients with histologically confirmed NETs of different primary tumor sites who received everolimus between August 2011 and October 2020 at our department. Eligible patients had liver metastases only or, if not already resected, the primary tumor and had undergone both baseline and follow-up ^68^Ga-DOTA-TATE (-TOC) PET/CT scans. The selection for everolimus therapy was based on consensus decisions made in an interdisciplinary tumor conference certified for NETs (ENETS Center of Excellence). The study received approval from the local research ethics committee, and the requirement for written informed patient consent was waived.

### PET/CT

Whole-body PET/CT scans were conducted using either a GE Discovery 690 (GE Healthcare, Little Chalfont, United Kingdom) or a Biograph 64 TruePoint PET/CT scanner (Siemens Healthcare, Erlangen, Germany). Approximately 60 minutes after intravenous injection of around 220 MBq of ^68^Ga-DOTA-TATE (-TOC), emission data were acquired. When feasible, 20mg of furosemide were administered. The emission data underwent reconstruction with attenuation correction using concurrent diagnostic CT scans, which covered the neck, thorax, abdomen, and pelvis. The diagnostic CT scan parameters were set at 100–190 mAs, 120 kV, with a collimation of 2 x 5 mm and a pitch of 1.5. Additionally, an iodine-based contrast agent (Ultravist 300TM; Bayer Healthcare, Berlin, Germany; 1.5 mL/kg body weight) was intravenously injected at a rate of 2.5 mL/s, with a 50-second delay to capture the portal venous phase of the liver.

### Image analysis

Two board-certified radiologists with experience in nuclear medicine, blinded to the patients’ clinical and follow-up data, conducted a consensus review of pre- and post-treatment PET/CT scans. Target lesions, including up to two liver metastases and the pancreatic NET if not resected, were identified based on criteria such as increased tracer uptake exceeding normal physiological activity and a minimum size of 1 cm, detectable on CT scans. In two separate reading sessions, the radiologists measured the maximum lesion size and density (in Hounsfield units) of the normal liver and spleen, as well as the liver metastases and NET, on CT scans. The uptake of ^68^Ga-DOTA-TATE was quantified by obtaining the maximum and mean standardized uptake values (SUVs). Circular volumes of interest (VOIs) were positioned within the predefined target lesion and in the normal liver and spleen parenchyma to calculate tumor-to-organ ratios, including the tumor-to-spleen (T/S) ratio and tumor-to-liver (T/L) ratio (using SUVmax/SUVmax, SUVmax/SUVmean, SUVmean/SUVmax and SUVmean/SUVmean).

### Standard of reference and response to treatment

Diagnosis of NET was confirmed by histopathology, and Ki-67 labelling index of the primary tumor was obtained for all included patients. Tumor grading was rated according to 2017 WHO Tumor Classification Guideline (G1: Ki-67 Index was <3%, G2: Ki-67 Index was 3–20%, and G3 NET/NEC: Ki-67 Index was >20%).^17^

Progression-free survival (PFS) was used as a parameter for treatment response, calculated in days/months from the time of everolimus initiation until progression, as evaluated by the local interdisciplinary tumor board through the assessment of all performed imaging studies (CT, PET/CT, MRI). Responder (R) were defined as patients with PFS > 6 months and non-responders (NR) as patients with PFS ≤ 6 months respectively.

### Statistical analysis

Statistical analysis was conducted using commercially available software, including GraphPad Prism Version 6 (San Diego, Calif.), SPSS version 25 (Chicago, IL), and Microsoft Excel v. 16. The level of statistical significance was defined as p ≤ 0.05. Data were presented as mean with standard deviation (SD) or median values with interquartile range [IQR], as appropriate. Normal distribution of continuous variables was assessed through visual inspection of the frequency distribution (histogram).

Quantitative measurements of the target lesions before and after therapy were compared using a two-tailed, paired t-test, while comparisons of target lesions between different response groups were performed using a two-tailed, unpaired t-test or Wilcoxon rank sum test, depending on the data distribution.

Progression-free survival (PFS) was analyzed using the Kaplan-Meier method, and survival curves were compared using the Log-Rank test. Prognostic parameters for PFS were assessed using Cox proportional hazards regression analysis. In the multivariable model, variables with a p-value ≤ 0.05 in the univariable analysis were included using a stepwise approach.

## Results

### Patients

A total of 29 patients (12 female, 17 male) with a combined count of 62 target lesions (54 liver metastases, 8 primary tumors) were included in this retrospective study. The baseline PET/CT scans were acquired 19 d (interquartile range (IQR) 69d) before treatment initiation, and the first follow-up PET/CT scans were performed 127 d (IQR 74d) after the start of everolimus treatment. Most of the included patients had G2 tumors (n = 20), followed by G1 tumors (n = 7) and one patient had a high-grade primary tumor (G3), while grading information was not available for one patient. Detailed patient characteristics are presented in Table 1.

**TABLE 1.** Patients demographics

Sex (male)	17 (59%)
Age (mean + SD )	63 ± 13.2
Grading	
G1	7 (24%)
G2	20 (69%)
G3	1 (3%)
n/a	1 (3%)
Ki-67 (mean + SD)	9.1 ± 7.6
Primary tumor site	
Pancreas	11 (38%)
Stomach	1 (3%)
Liver	1 (3%)
Lung	1 (3%)
Small-intestine	11 (38%)
Kidney	1 (3%)
Breast	1 (3%)
Retroperitoneal	1 (3%)
Rectum	1 (3%)
Bilirubin prior to therapy (mg/dl)	0.6 (0.3–1.3)
Bilirubin after therapy (mg/dl)	0.5 (0.2–2.6)
Median CgA prior to therapy (ng/ml) (range)	487 (10 –8983)
Median CgA after therapy (ng/ml) (range)	929 (43–23348)

CgA = chromogranin A; SD = standard deviation

### Progression-free survival

At the end of the study, 28/29 patients showed progression on imaging. The overall median PFS was 264 days (95% CI: 134–394 days). The median PFS was 487 days (95% CI: 154–820 days) in the R group (n = 16), and 112 d in the NR group (95% CI: 79–145 days). There was no significant difference in PFS between patients with elevated and non-elevated baseline chromogranin A (CgA) levels (p > 0.7).

### Responder *vs*. non-responder

**FIGURE 1.**
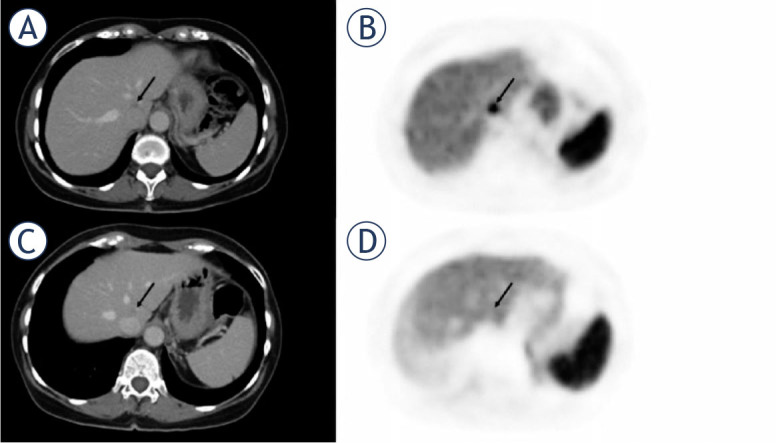
63-year-old female with responding liver metastasis of ileal neuroendocrine tumor. On the pretherapeutic PETCT **(A, B)** there were high tumor-to-liver (T/L) ratio. After three months of treatment with everolimus, the liver metastasis showed no significant shrinkage in size, but a significantly reduced uptake of ^68^Ga-DOTATATE **(C, D)** compared to pretherapeutic PET/CT (Tmax/Lmax: 0.94 *vs*. 2,09 and Tmean/Lmax 1.54 *vs*. 0.76).

**FIGURE 2.**
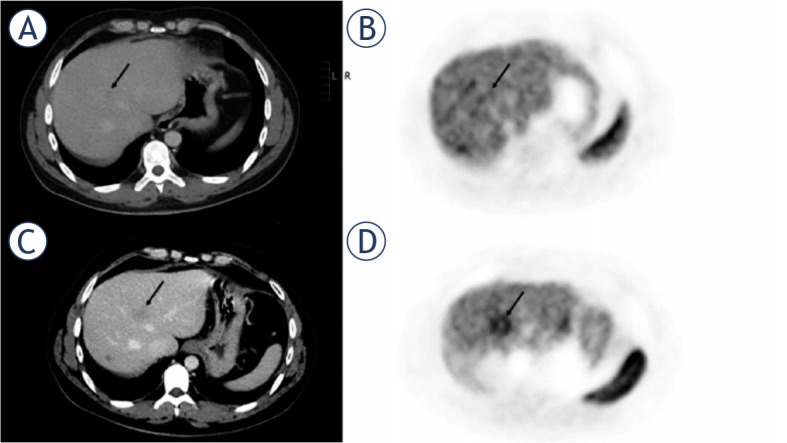
31-year-old male with non-responding liver metastasis of pancreatic neuroendocrine tumor. On the pretherapeutic PET/CT **(A, B)** there were low tumor-to-liver (T/L) ratios. After three months of treatment with everolimus, liver metastasis (arrow) showed an increase in size on CT **(C)**, but also a significantly increased uptake of ^68^Ga-DOTATATE **(D)** compared to pretherapeutic PET/CT.

There were no differences in pretherapeutic clinical or imaging parameters between the two response groups. In responders (R), the absolute SUV of the liver metastases (including SUVmax and SUVmean) decreased significantly, while there was no change in non-responders (NR). However, the percentage changes between the response groups were not significantly different. Tmax/Lmax and Tmean/Lmax of liver metastases decreased significantly in responders, while there was no change in non-responders, and the percentage changes were significantly different, with −15.5% in responders compared to 5.5% in non-responders (p = 0.01) (Figures 1 and 2). There were no significant changes in the size of the liver metastases. The density of the liver metastases decreased significantly in responders, while there was no change in non-responders; however, percentual changes were not different between both response groups.

Regarding the primary NET, statistical evaluation was limited due to the small sample size and different primary tumor sites (pancreas: n = 6, duodenum: n = 1, rectum: n = 1), and there were no significant changes in response groups.

SUVmean of the spleen increased significantly in responders (p = 0.02), while it decreased in non-responders (P = 0.04). SUVmax of the liver decreased in both responders and non-responders, while the percentage decrease was slightly higher in non-responders (p = 0.04) (Table 2).

**TABLE 2.** Clinical and imaging parameters at baseline and follow-up with changes

	**Responder**		**Pre- vs. Post-therapy**	**Non-responder**		**p-value**	**R vs. NR Pre-treatment**	**R vs. NR Post-treatment**
	
	**Pre-treatment**	**Post-treatment**	**p**	**Pre-treatment**	**Post-treatment**		**p**	**p**
**Clinical parameters**								
Age	63.5 +- 12.5			62.4 +- 13.4			0.42	
Sex (male)	10 (45%)			7 (37%)			0.2	
Ki-67 %	7.9 (+- 7.8)			10.5 (+-7)			0.28	
Pre Bilirubin	0.6 (0.2)			0.7 (0.3)			0.13	
Pre CgA	551 (77.6–933.5)			422 (47–1414)			0.11	
**Imaging parameters**								
SUVmax Liver	8 (6–9.3)	9.4 (5–10.9)	0.17	6.9 (4.9–9.2)	6.4 (3.5–10)	0.05	0.6	0.1
SUVmean Spleen	12.3 (9.9–18.6)	16.4 (9.9–20.1)	**0.02**	13.8 (10.1–16.9)	11 (9.8–15.1)	**0.04**	0.3	0.5
SUVmax LM	28.1 (15.1–35.3)	22.2 (14.2–31.7)	**< 0.01**	21.3 (12.7–34.7)	22.5 (9.4–29.6)	0.19	0.3	0.6
SUVmean LM	12.4 (10.5–19.1)	12.9 (9.3–18.9)	**0.03**	14.5 (9.3–18.3)	13.5 (7.8–17.4)	0.13	0.4	0.5
Tmax/Lmax LM	3.0 (2.4–4.3)	2.4 (2–3.3)	**0.02**	2.8 (2.4–3.8)	3 (1.9–4.6)	0.36	0.3	0.8
Tmean/Lmax LM	1.8 (1.4–2.3)	1.6 (1.2–1.9)	**0.02**	1.9 (1.2–2.2)	1.6 (1.4–2.5)	0.26	0.2	0.7
Tmax/Smean LM	1.6 (1.3–2.4)	1.3 (0.9–1.7)	0.25	1.5 (1.2–2.3)	1.5 (0.9–3.0)	0.3	0.4	0.8
Tmean/Smean LM	1.0 (0.8–1.1)	0.8 (0.5–1.0)	0.49	0.9 (0.6–1.6)	0.9 (0.5–1.6)	0.46	0.5	1.0
Size LM	21 (17–29)	20 (15–31.2)	0.64	21 (15–32)	21 (15–32)	0.95	0.7	0.5
Density LM (HU)	101.9 (± 19.9)	90.5 (± 20.5)	**0.03**	89.4 (± 22.6)	81.7 (± 22.1)	0.1	0.2	0.3
SUVmax NET	32.8 (6.9–39.8)	30.4 (10.3–31.5)	0.49	36.4 (28.8–47.4)	43.1 (29.7–53.7)	0.97	0.5	0.3
SUVmean NET	16 (4.6–16.9)	13.4 (7.1–14.7)	0.9	17.6 (14–19.4)	22.3 (13.6–28.5)	0.25	0.4	0.3
Size NET	18 (15-36)	25 (11.2–37.3)	0.48	43.5 (24–60.8)	44.5 (25.3–66)	0.37	0.7	0.5
Density NET	83.3 (± 9.1)	81.5 (± 18)	0.9	89.3 (± 17.5)	78.2 (± 11.6)	0.4	0.6	0.8
**Change (%) between pre- and post-treatment**	**Responder**			**Non-responder**		**R vs. NR p**		
SUVmax Liver	−9.5 (−14–19.9)			−13 (−37.5–8.7)		**0.04**		
SUVmean Spleen	13 (−6.6–29.1)			−10.7 (−26.3–2.6)		**0.01**		
SUVmax LM	−20.4 (−27–6)			−0.9 (−22.8–26.3)		0.21		
SUVmean LM	−10 (−28.5–12.7)			−7 (−23.4–11.1)		0.62		
Tmax/Lmax LM	−15.5 (−47–5.5)			5.5 (−20.4–20.7)		**0.01**		
Tmean/Lmax LM	−16.3 (−37–9)			7 (−22.3–31.1)		**0.03**		
Tmax/Smean LM	−16.6 (−44.2–13.6)			4.5 (−16.8–51.4)		**0.01**		
Tmean/Smean LM	−16.3 (−39.3–8.8)			0.9 (−17.4–50)		**0.04**		
Size LM	−7 (−27.8–6.1)			14.3 (−17.6–28)		0.77		
Density LM	−5.6 (−12.3–0.2)			−7 (−16.5–4.1)		0.8		
SUVmax NET	−7.3 (−16.2–35.5)			−18.1 (−41.6–25.6)		0.77		
SUVmean NET	15.6 (−0.6–48)			18.4 (−7.8–40.5)		0.65		
Size NET	−15.9 (−37.4–11.1)			3.6 (−2.1–13.3)		0.72		
Density NET	−5.2 (−20–14.5)			−18.3 (−22.9– −4.6)		0.69		
Bilirubin	−33 (−61.7–0)			−25 (−66.7–0)		0.43		
CgA	56.4 (17.4–144.9)			32.1 (−2.8–72.8)		0.18		

CgA = chromogranin A; LR = liver metastases; NET = neuroendocrine tumor; NR = non-responder; R = responder; SUV = standardize uptake value; Tmax/Lmax = tumor max to liver max; Tmax/Smean = tumor max to splen max

### Prognostic and predictive factors

In the univariate Cox regression analysis, the only prognostic parameter associated with PFS was the Tmean/Lmax ratio of the liver metastases (HR 0.5, 95% CI 0.28–0.92, p = 0.03). Patients with a higher Tmean/Lmax ratio (≥ 2) on baseline imaging showed slightly significantly longer PFS, with a median of 410 days compared to 185 days (p = 0.04). None of the pretherapeutic clinical parameters were associated with PFS.

**FIGURE 3.**
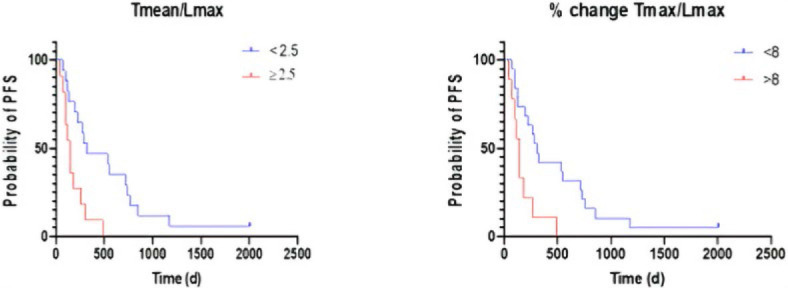
Predictive value of %-change of Tmean/Lmax ratio and %-change of Tmax/Lmax ratio.

Among the changes after treatment, the percentage changes of both tumor-to-liver ratios (Tmax/Lmax and Tmean/Lmax) and the Tmax/Smean ratio were significant predictors of PFS. In a secondary analysis, the area under the curve (AUC) was highest for the percentage change of Tmean/Lmax (AUC = 0.73), followed by the percentage change of Tmax/Lmax (AUC = 0.71). The ROC analysis revealed an optimal threshold for Tmean/Lmax at >2.5 (sensitivity 62%, specificity 80%) and for Tmax/Lmax at >8 (sensitivity 54%, specificity 87%). PFS of patients with any decrease of Tmax/Lmax less than 2.5% was significantly longer with 322 days compared to 142 days (p = 0.003). Also, PFS of patients with an any decrease of Tmax/Lmax less than 8% was significantly longer with 306 days compared to 142 days (p = 0.005) (Figure 3). None of the other imaging parameters, such as density or size, nor any of the clinical parameters, were predictive of PFS.

## Discussion

In this study we investigated the utility of clinical, morphological, and SSTR-PET derived functional imaging parameters for evaluating response and predicting outcomes in NET patients treated with everolimus. Our findings suggest that the use of ratios of SUV of the liver metastases (Tmean/Lmax and Tmax/Lmax) could serve as valuable tool for assessing and even predicting therapy response in NET patients receiving everolimus.

We chose progression-free survival (PFS) as the primary endpoint for response evaluation, following the recommendation of the National Cancer Institute Neuroendocrine Tumor Clinical Trials Planning Meeting consensus report. The use of overall survival (OS) for therapy response evaluation in NETs can be challenging due to the long survival times typically associated with these tumors and the range of post-progression salvage therapies available.^18^ The overall median PFS in our patient cohort was slightly lower compared to the RADIANT-3 and RADIANT-4 trials, with 8.7 months compared to 11 months, respectively.^5,19^ These differences could be attributed to factors such as the small size of our study cohort, the use of everolimus as a late treatment-line at our clinic, and the distribution of tumor grading among patients. In our patient cohort 69% had G2 tumors and 24% had G1 tumors, while in the RADIANT-3 study 82% of the patients had well differentiated tumors and in the RADIANT-4 study 63% had G1 tumors.^5,19^

Conventional response assessment based on percentage changes in the size of target lesions did not show significant differences between the response groups on the first follow-up PET/CT. However, functional imaging parameters, particularly the percentage changes in tumor-to-organ ratios of the liver metastases, were significantly different between response groups, with non-responders showing a median percent increase (median 0.9 to 7%) and responders showing a median percent decrease (median −15.5 to −16.6%). The percentual changes of absolute SUV measurements however, including SUVmax and SUVmean, were not significantly different between response groups.

Tumor-to-organ ratios are affected by the changes in the specific organs, e.g., liver or spleen respectively. Our study revealed a significant increase in spleen SUVmean after treatment in R, while NR showed a decrease in splenic SUVmean. Previous studies have reported a decrease in SSTR uptake in the spleen after long-acting somatostatin analog therapy, accompanied by an increase in tumor uptake.^20,21^ Additionally, after splenectomy, higher uptake of ^68^Ga DOTA-TOC in tumors and some normal tissues has been observed.^22,23^ However, these changes’ correlation with tumor response has rarely been evaluated.

Quantitative assessment of SSTR PET/CT has its challenges, and relying solely on absolute tumor uptake for response evaluation might lead to erroneous conclusions.^20^ In this context, tumor-to-organ ratios offer an advantage, along with the normalization of absolute SUV to obtain scanner-independent parameters. In our study, SUVmax of the liver decreased in both responders and non-responders, with a slightly greater degree of reduction in non-responders (−13% *vs*. −9.5%). This observation could be related to the metabolization of everolimus, which mainly occurs in the liver via the CYP3A4 system. Although serum enzyme elevations may occur in up to 25% of patients, these changes are typically mild and self-limiting.^24,25^

In a secondary analysis, we evaluated the association of different clinical and imaging parameters with PFS using Cox regression analysis. Among the prognostic factors, the Tmean/Lmax ratio of the liver metastases emerged as the only significant parameter associated with PFS (HR 0.5, 95% CI 0.28–0.92, p = 0.03). Patients with a higher Tmean/Lmax ratio (≥ 2) on baseline imaging exhibited longer PFS, while none of the pretherapeutic clinical parameters showed a significant association with PFS. These findings are consistent with previous studies investigating the prognostic value of SSTR-PET/CT in patients undergoing peptide-receptor-radionuclide therapy (PRRT)^26,27,28^ or lanreotide treatment^29^, which also reported that patients with higher baseline tumor-to-liver ratios or SUVmax had a better prognosis or were more likely to respond to therapy. For PRRT, Kratochwil *et al*. proposed a cut-off value of the tumor-to-liver ratio > 2.2 to select patients for treatment by PRRT.^27^ The correlation between higher SUV values and the dose delivered by PRRT may explain these findings. However, in the context of a targeted therapy like everolimus, the underlying mechanism remains unclear. One possible explanation is that a target lesion with higher SSR expression might correspond to a more differentiated tumor. Further research is needed to elucidate the precise mechanisms underlying these observations.

In the univariable Cox regression analysis, the percentage change of the tumor-to-organ ratios (Tmax/Lmax and Tmean/Lmax) were found to be significantly associated with PFS. However, none of these parameters remained significant in the multivariable analysis, possibly due to the total study size being a limiting factor for this analysis. ROC analysis was performed on the identified parameters, and the highest AUC was obtained for Tmean/Lmax (AUC = 0.73). Patients with a percentual change of Tmax/Lmax of less than 2.5% showed a significantly longer PFS with 322 days compared to 142 days (p = 0.003). In contrast, none of the other imaging parameters, such as density or size, nor any of the clinical parameters, were predictive of PFS at the first follow-up.

Increasing evidence suggests that conventional response assessment based on tumor size change is limited in evaluating treatment response to anti-proliferative or antiangiogenic effects mediated by targeted anti-cancer drugs, particularly in slow-growing tumors such as NETs.^8,9,10^ Low objective response rates in the RADIANT 3 and RADIANT 4 trials, with 5% and 2% in the everolimus group, respectively, further support this observation.^5,19^ It suggests that the benefit of everolimus on PFS is mainly attributed to the stabilization of tumor growth or minor tumor shrinkage, without reaching the cutoff for partial response as defined by RECIST.^5,8^ Consequently, conventional size-based response criteria in this context may not accurately classify patients as responders *vs*. non-responders, potentially leading to the inappropriate discontinuation of an effective therapy.^21^

For classical FDG PET/CT, numerous studies have demonstrated that a decrease in SUV after therapy can predict survival. For instance, in patients with liver metastasis of pancreatic cancer treated with TARE or in breast cancer patients receiving targeted therapies, a decrease in SUV has been shown to be indicative of improved outcomes.^30,31^ However, data regarding SSTR PET/CT is more limited. Some studies have indicated that a decrease in SUV, corrected for spleen or liver uptake, is associated with longer time to progression after PRRT and even correlated with improved clinical symptoms.^32,33^ Additionally, in a different study, the change of T/S and T/L ratios on the first follow-up was identified as the best metric to correlate with longer hepatic PFS in NET patients undergoing TARE.^34^

The main limitation of this study was its small sample size, which is related to the low incidence of NETs and everolimus representing a second-line treatment. Additionally, the retrospective analysis represents a limiting factor, as time intervals between PET/CT scans and everolimus treatment were heterogeneous, and prior therapies differed among patients. Another limitation is the use of different scanners. While the use of quantitative SUV is well-established for FDG PET/CT using the PERCIST criteria^35^, the interpretation of SUV in SSTR-PET/CT is more complex, as a reduction in uptake could be attributed to tumor regression or dedifferentiation.^26^ Therefore, the use of normalized SUV measures by calculating tumor-to-spleen, liver, or blood pool ratios was suggested by various authors.^33,36^

In conclusion, our study highlights the potential value of quantitative SSTR-PET/CT in predicting and monitoring response in patients with NETs receiving everolimus. The use of liver metastasis-to-liver parenchyma ratios (Tmean/Lmax and Tmax/Lmax) showed promise in assessing therapy response, outperforming conventional size-based criteria. The Tmean/Lmax ratio emerged as a promising prognostic marker for progression-free survival (PFS), warranting further investigation in larger cohorts. Despite limitations of retrospective nature and small sample size, functional imaging remains crucial for guiding personalized treatment strategies in NET patients undergoing targeted therapies like everolimus. Prospective studies with standardized protocols and larger populations are needed to validate these findings’ clinical relevance.
